# Targeting survivin as a potential new treatment for chondrosarcoma of bone

**DOI:** 10.1038/oncsis.2016.33

**Published:** 2016-05-09

**Authors:** Y de Jong, J G van Oosterwijk, A B Kruisselbrink, I H Briaire-de Bruijn, G Agrogiannis, Z Baranski, A H G Cleven, A-M Cleton-Jansen, B van de Water, E H J Danen, J V M G Bovée

**Affiliations:** 1 Department of Pathology, Leiden University Medical Center, Leiden, The Netherlands; 2 First Department of Pathology, Department of Clinical-laboratory Studies, Athens University Medical School, Athens, Greece; 3 Division of Toxicology, Leiden Academic Center for Drug Research, Leiden University, Leiden, The Netherlands

## Abstract

Chondrosarcomas are malignant cartilage-forming bone tumors, which are intrinsically resistant to chemo- and radiotherapy, leaving surgical removal as the only curative treatment option. Therefore, our aim was to identify genes involved in chondrosarcoma cell survival that could serve as a target for therapy. siRNA screening for 51 apoptosis-related genes in JJ012 chondrosarcoma cells identified *BIRC5*, encoding survivin, as essential for chondrosarcoma survival. Using immunohistochemistry, nuclear as well as cytoplasmic survivin expression was analyzed in 207 chondrosarcomas of different subtypes. Nuclear survivin has been implicated in cell-cycle regulation while cytoplasmic localization is important for its anti-apoptotic function. RT–PCR was performed to determine expression of the most common survivin isoforms. Sensitivity to YM155, a survivin inhibitor currently in phase I/II clinical trial for other tumors, was examined in 10 chondrosarcoma cell lines using viability assay, apoptosis assay and cell-cycle analysis. Survivin expression was found in all chondrosarcoma patient samples. Higher expression of nuclear and cytoplasmic survivin was observed with increasing histological grade in central chondrosarcomas. Inhibition of survivin using YM155 showed that especially *TP53* mutant cell lines were sensitive, but no caspase 3/7 or PARP cleavage was observed. Rather, YM155 treatment resulted in a block in S phase in two out of three chondrosarcoma cell lines, indicating that survivin is more involved in cell-cycle regulation than in apoptosis. Thus, survivin is important for chondrosarcoma survival and chondrosarcoma patients might benefit from survivin inhibition using YM155, for which *TP53* mutational status can serve as a predictive biomarker.

## Introduction

Chondrosarcoma is a malignant cartilage-forming tumor accounting for 20% of all malignant bone tumors.^1^ Chondrosarcomas represent a heterogeneous group as different histological subtypes are recognized, including conventional, dedifferentiated, mesenchymal, clear cell and periosteal chondrosarcoma. The conventional subtype is most frequent (85%)^1^ and can be further categorized into central chondrosarcoma (>85%) (in medullar cavity) and peripheral chondrosarcoma (at the bone surface).^2^ Histologically, atypical cartilaginous tumors (ACT) (previously referred to as grade I chondrosarcomas) show low cellularity and are locally aggressive, but do not metastasize. High-grade chondrosarcomas comprise grade II and III chondrosarcomas and show higher cellularity, mitoses and less cartilaginous matrix. Histological grading represents the most important prognostic factor; patients with atypical cartilaginous tumors show a 10-year overall survival rate of 83%, patients with grade II tumors show 64% survival and patients with grade III chondrosarcomas show 29% 10-year survival rate.^1,2^ Dedifferentiated chondrosarcomas comprise 10% of all chondrosarcomas and are characterized by a high-grade dedifferentiated component juxtaposed to a low-grade cartilaginous component. Patients with dedifferentiated chondrosarcoma show a 5-year overall survival between 7 and 24%.^3^ Mesenchymal chondrosarcoma is a rare (<3%) high-grade chondrosarcoma subtype with reported 10-year survival rates between 27 and 67%.^4,5^ Histologically, it consists of differentiated cartilage mixed with undifferentiated small round cells.^6^

Chondrosarcomas are intrinsically resistant to conventional chemo- and radiotherapy, and therefore surgical removal of the tumor is the only curative treatment option. Several studies have been performed investigating possible new therapeutic targets for the treatment of chondrosarcoma. This has led to several discoveries including mTOR,^7,8^ Src^9,10^ and Bcl-2 family members^11,12^ as possible targets but still no targeted therapies are available for chondrosarcoma patients. Therefore, there is still an urgent need for novel therapeutic targets that can be easily and rapidly applied in the treatment of patients with high-grade metastatic or inoperable chondrosarcoma.

To identify new cancer drug targets, high-throughput RNA interference (RNAi) screens are widely used and have led to several important findings regarding new cancer gene discoveries.^13^ Loss of function RNAi screens have led to the identification of important oncogenes in several different cancer types, such as the identification of Brd4 as a therapeutic target in acute myeloid leukemia^14^ and MED12 as a determinant of drug response to tyrosine kinase inhibitors in non-small cell lung cancer.^15^ As RNAi is a powerful tool to discover survival-related genes in a specific manner, we performed an siRNA screen targeting 51 genes involved in apoptosis regulation to identify genes involved in survival of chondrosarcoma cells that could serve as a potential target for therapy for patients with inoperable or metastatic chondrosarcoma. To validate our results, we used a unique panel of chondrosarcoma cell lines including conventional, dedifferentiated and mesenchymal subtypes, reflecting the heterogeneity of chondrosarcoma of bone.

## Results

### BIRC5 is an essential survival gene in chondrosarcoma cells

**Figure 1:**
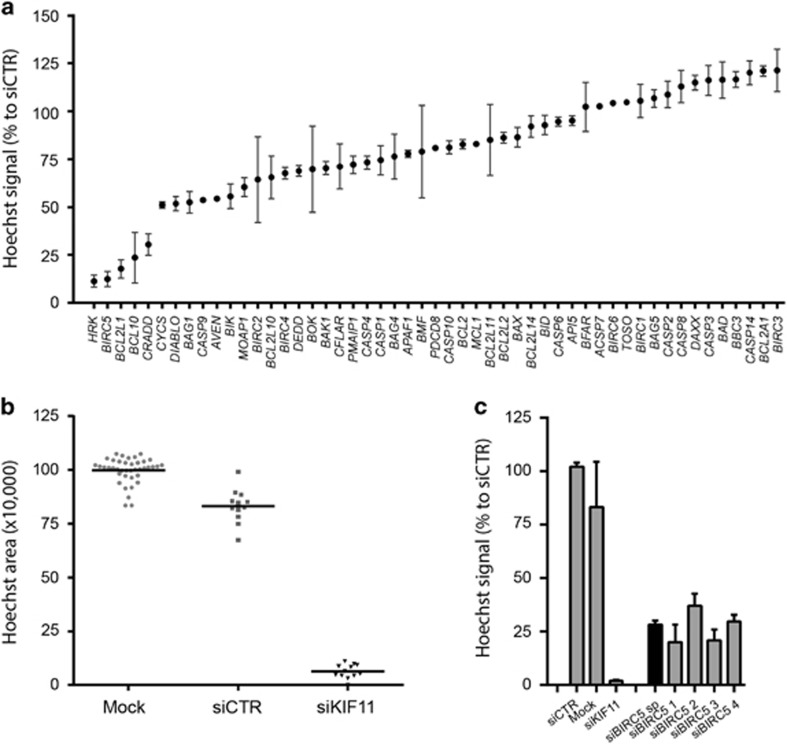
Screening for apoptotic regulators identifies *BIRC5* as an important survival gene in chondrosarcoma cell line JJ012. (**a**) Occupied area of cells present in the well as a percentage to control siRNA of 51 apoptosis-related genes. Mean values and range of duplicates are shown. (**b**) Raw data measured as Hoechst area of controls used in the siRNA screen. Dots indicate individual measurements and mean values are shown for each control. siCTR indicates control siRNA of either GFP or GAPDH. (**c**) Area of cells as a percentage to control siRNA of selected hits. *BIRC5* shows 4/4 siRNAs that mimic the smart pool. Data represent means of duplicate values with range. Black bars represent the smart pool and gray bars represent individual siRNAs.

An siRNA screen targeting 51 apoptosis-related genes revealed that *HRK* (Harakiri, BCL2 Interacting Protein), *BIRC5* (Baculoviral IAP Repeat Containing 5), *BCL2L1* (B-cell/Lymphoma 2 like 1), *BCL-10* (B-cell/Lymphoma 10) and *CRADD* (CASP2 And RIPK1 Domain Containing Adaptor With Death Domain) were essential genes in the JJ012 chondrosarcoma cell line (Figure 1a). Across the primary screen, transfection with control siRNAs led to a slight (~13%) reduction in viability compared with mock whereas positive control si*KIF11* caused a very strong (>90%) loss of viability indicating successful gene silencing and a good experimental window (Figure 1b). The average Z'factor was 0.61, which indicates a qualitatively good screen. Deconvolution was performed for selected hits, and *BCL2L1*, *BIRC5*, *CRADD* and *HRK* were confirmed (Supplementary Figure 1). Identification of *HRK* and *CRADD* is surprising as these encode pro-apoptotic proteins. A role for *BCL2L1* in chondrosarcoma has been previously described by us.^11^ Here, we focused on *BIRC5* (Figure 1c), which encodes the survivin protein, an anti-apoptotic protein overexpressed in human cancers.^16^

### Survivin is highly expressed in chondrosarcoma tumor tissue and cell lines

**Figure 2:**
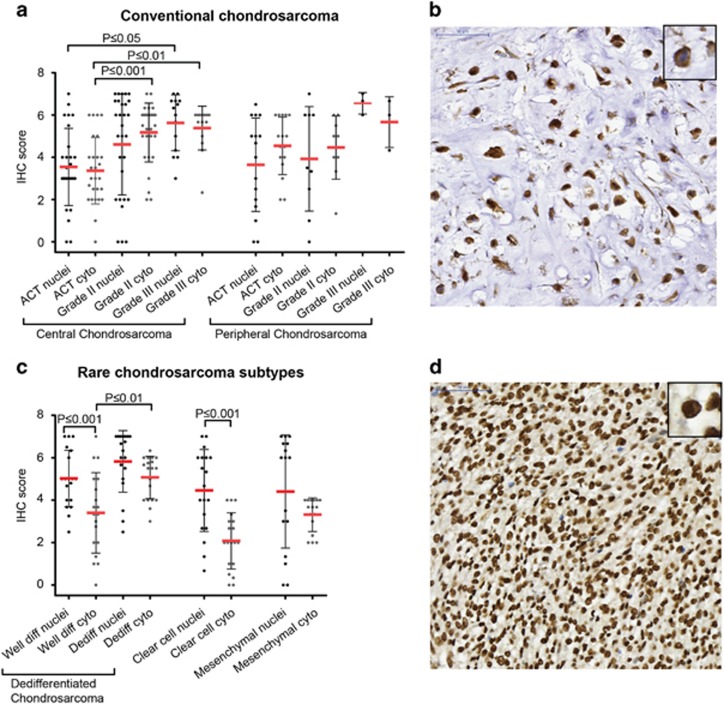
Survivin is highly expressed in high-grade chondrosarcoma. (**a**) Immunohistochemical analysis of survivin in conventional chondrosarcoma showing higher cytoplasmic expression in grade II and III central chondrosarcoma compared with ACTs. Also higher expression of nuclear survivin is observed in grade III chondrosarcomas compared with ACTs. Each dot represents one patient, and mean value with standard deviation is shown for each group. (**b**) High cytoplasmic survivin expression in a grade III chondrosarcoma. (**c**) Immunohistochemical analysis of nuclear and cytoplasmic survivin in dedifferentiated, clear cell and mesenchymal chondrosarcoma. Nuclear survivin is highly expressed in nuclei of the well-differentiated part of dedifferentiated chondrosarcoma and clear cell chondrosarcoma compared with the cytoplasmic part. Each dot represents one patient, and mean value with standard deviation is shown for each group. (**d**) High nuclear survivin expression in dedifferentiated chondrosarcoma (dedifferentiated part).

Survivin protein expression and its subcellular localization were determined in 207 chondrosarcomas of different subtypes. Nuclear expression, suggesting a role for survivin in cell-cycle progression, was found in 90/98 conventional chondrosarcomas. Cytoplasmic expression, reflecting its function in apoptosis regulation, was found in 97/98 conventional chondrosarcomas. Expression levels were variable. Both nuclear and cytoplasmic survivin expression increased with increasing histological grade in central chondrosarcoma (Figures 2a and b). Cytoplasmic survivin expression was significantly higher in grade II (*P*⩽0.001) and III (*P*⩽0.01) central chondrosarcomas compared with ACTs. Also, higher expression of nuclear survivin was observed in grade III chondrosarcomas compared with ACTs (*P*⩽0.05). No correlation was found with *IDH1* or -*2* mutation status (not shown). Moreover, survivin was also highly expressed in dedifferentiated, clear cell and mesenchymal chondrosarcoma. Survivin expression was significantly higher in nuclei compared with the cytoplasm in the well-differentiated part of dedifferentiated chondrosarcoma (*P*⩽0.001) (Figures 2c and d). Also in clear cell chondrosarcoma, nuclear expression was significantly higher (*P*⩽0.001) compared with cytoplasmic expression (Figure 2c).

### Nuclear survivin expression is associated with p53 overexpression in high-grade conventional chondrosarcoma

**Figure 3:**
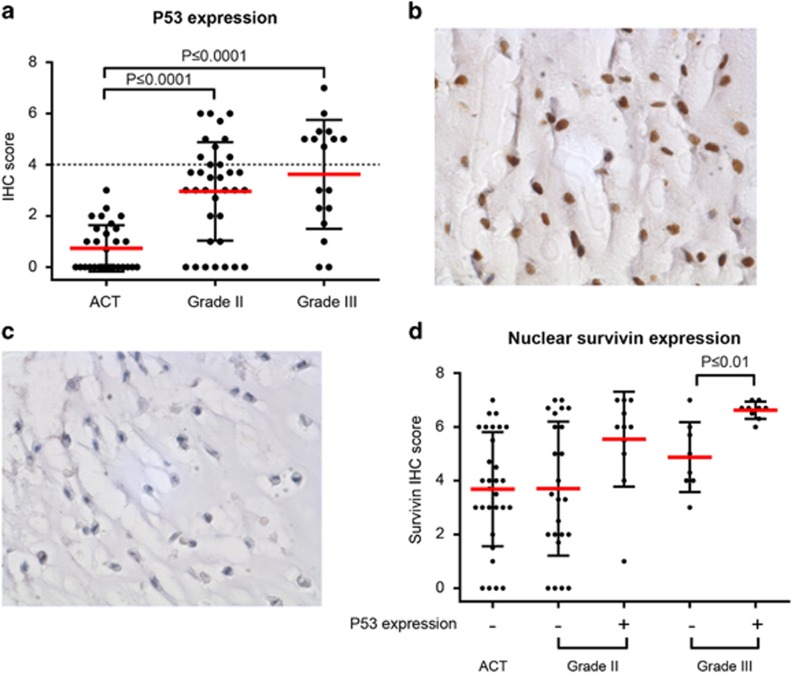
P53 is highly expressed in high-grade conventional chondrosarcoma and correlated with nuclear survivin in grade III chondrosarcoma. (**a**) Immunohistochemical analysis of p53 expression in conventional chondrosarcoma. Grade II and III chondrosarcomas show significantly higher expression compared with grade I chondrosarcoma (*P*<0.0001). Each dot represents one patient, and mean value with standard deviation is shown for each group. (**b**) High nuclear p53 expression in a high-grade chondrosarcoma. (**c**) Low nuclear p53 expression in a high-grade chondrosarcoma. (**d**) Correlation between nuclear survivin and p53 overexpression in conventional chondrosarcoma. P53 is considered as overexpressed when it reached a sum score of 4 (indicated by +). Grade I chondrosarcomas did not show p53 overexpression. In grade II chondrosarcoma, higher nuclear survivin was seen in p53 overexpressing chondrosarcomas, as well as in grade III chondrosarcoma (*P*<0.01) compared with low p53 expressing tumors. Each dot represents one patient, and mean value with standard deviation is shown for each group.

As p53 is a known effector of survivin expression,^17^ we investigated a possible correlation between p53 overexpression (indicative of a mutated *TP53* gene) and survivin expression using the conventional chondrosarcoma tissue micro array (TMA). p53 overexpression was observed in 0/50 grade I, 20/41 grade II and 9/17 grade III conventional chondrosarcomas (Figures 3a and b). In high-grade chondrosarcomas (grade II and III) with overexpression of p53, the expression of nuclear survivin was higher as compared with high-grade chondrosarcomas without overexpression of p53 (*P*⩽0.01 for grade 3) (Figure 3c). No difference between peripheral and central conventional chondrosarcoma was observed (data not shown).

### Survivin isoforms are highly expressed in high-grade chondrosarcoma

**Figure 4:**
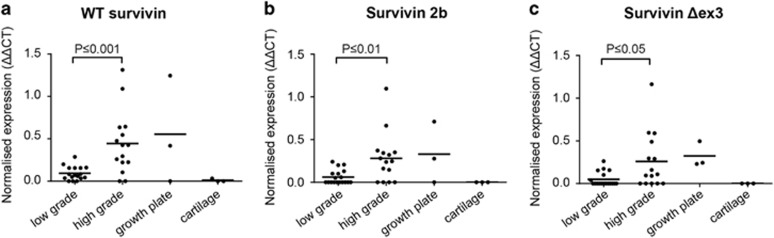
Survivin wt, 2b and Δex3 isoforms are highly correlated and show higher expression in high-grade chondrosarcoma as determined by Q-PCR analysis. (**a**–**c**) Survivin isoforms wt (**a**), 2b (**b**) and Δex3 (**c**) are highly expressed in high-grade (grade II and III) chondrosarcoma compared with low-grade (ACT) chondrosarcoma. No expression is found in cartilage while variable expression is seen in growth plate tissue. Each dot represents one measurement, and mean values are shown for each group.

The three most common survivin isoforms, wild type (wt) survivin, survivin 2b and survivin Δex3, were all expressed significantly higher in high-grade chondrosarcomas (grade II and III) compared with low-grade chondrosarcomas (*P*⩽0.001 for wt survivin, *P*⩽0.01 for survivin 2b and *P*⩽0.05 for survivin Δex3) (Figures 4a–c). No significant difference in survivin expression was observed between *IDH* wt and *IDH1* or -*2* mutant chondrosarcomas (not shown). Furthermore, a correlation was found between all different survivin isoforms (Supplementary Figure 2).

### Chondrosarcoma cell lines highly express survivin, predominantly in the nucleus

**Figure 5:**
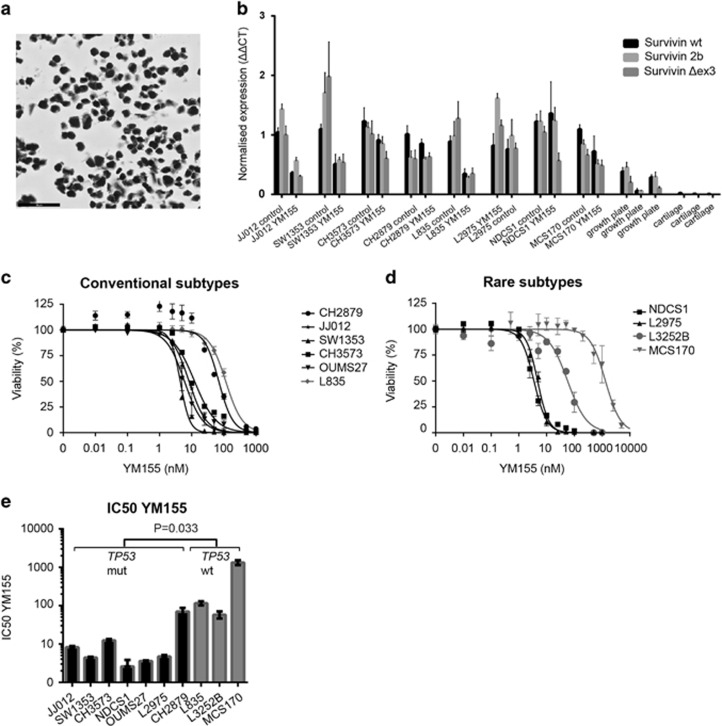
Chondrosarcoma cell lines are sensitive to YM155, which is p53 dependent. (**a**) JJ012 cell line showing strong survivin expression. (**b**) Normalized RNA expression of three survivin isoforms in eight chondrosarcoma cell lines. JJ012, SW1353 and L835 show a clear reduction in survivin expression after YM155 treatment. Growth plate and cartilage are taken as a control. Bars represent mean with standard deviation. (**c**, **d**) Dose response curves for YM155 (72 h) in conventional subtypes (**c**) and rare chondrosarcoma subtypes (**d**). Error bars are shown for three experiments performed in triplicate. (**e**) IC_50_s determined for *TP53* wt and mutant cell lines showing a significantly increased sensitivity of *TP53* mutant cell lines toward YM155. Bars represent mean values of three experiments performed in triplicate.

We confirmed that all cell lines showed high expression of survivin protein, predominantly in the nucleus (Figure 5a and Supplementary Figure 3). All survivin isoforms were highly expressed compared with normal articular cartilage (Figure 5b). Using the Ion AmpliSeqCancer Hotspot Panel v2, the *IDH1* or -2 mutation status was confirmed (Supplementary Table 1). For *TP53,* the use of this highly sensitive targeted next-generation sequencing technique provided novel insights for CH2879 and L2975, which were previously reported to be wild type (Supplementary Table 1).^18, 19, 20^ In CH2879, a subclonal pathogenic mutation was found with a frequency of 17%, which corresponded to the mosaic staining pattern of nuclear p53 observed in the cell pellet and primary tumor (Supplementary Figure 4). The mRNA expression levels of survivin were not correlated with *TP53* or *IDH1* or -*2* mutational status (not shown).

### Chondrosarcoma cell lines are sensitive to survivin inhibition using YM155

YM155 is a compound currently in phase I/II clinical trial that represses survivin promoter activity.^21^ We first assessed the effect of YM155 on survivin RNA downregulation (Figure 5b). JJ012, SW1353 and L835 cell lines showed clear downregulation of all three survivin isoforms after YM155 treatment. NDCS1 only showed downregulation of survivin Δex3 after YM155 treatment, and L2975 only showed downregulation of survivin 2b. Chondrosarcoma cell lines were highly sensitive to YM155 showing IC_50_ values below 5 nm in four out of ten chondrosarcoma cell lines (SW1353, OUMS27, NDCS1, L2975) (Figures 5c and d). JJ012 showed an IC_50_ of 8.2 nm and CH3573 showed an IC_50_ of 12.32 nm. The least responsive cell lines were L835, CH2879, L3252B and MCS170. Interestingly, three of these are *TP53* wt (*P*=0.033) (Figure 5e and Supplementary Table 1). There was no relation between YM155 sensitivity and histological subtype or *IDH1* or -*2* mutation status. Combination treatment of YM155 with doxorubicin and cisplatin did not show a synergistic effect in JJ012 and SW1353 cell lines (Supplementary Figure 5).

### The survivin inhibitor YM155 does not induce caspase 3/7 or PARP-dependent apoptosis in chondrosarcoma cells

**Figure 6:**
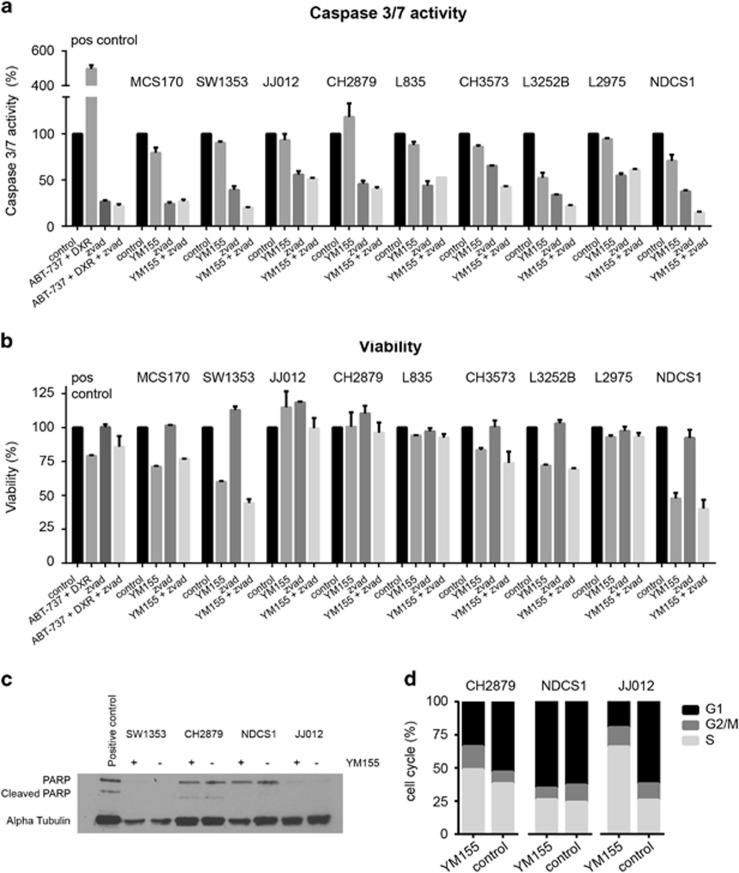
YM155 does not cause caspase 3/7 and PARP-dependent apoptosis, but cell-cycle deregulation in chondrosarcoma cell lines. (**a**, **b**) Caspase 3/7 activity (**a**) and viability (**b**) after 24 h as percentage to untreated control measured in nine chondrosarcoma cell lines by caspase-glo assay and presto blue viability assay. No caspase 3/7 activity is seen after YM155 treatment in all cell lines. Pan Caspase inhibitor z-vad was not able to rescue the YM155-dependent reduction in viability. MCS170 cells treated with doxorubicin and ABT-737 were used as a positive control. Error bars are shown for two independent experiments performed in duplicate. (**c**) Western blot analysis for PARP and cleaved PARP expression in four chondrosarcoma cell lines. No differences are seen between treated (+) and untreated (–) samples. Alpha tubulin was used as a loading control. Jurkat cell lysates treated with 25 μm etoposide obtained from Cell Signaling were used as a positive control. (**d**) FACS analysis of three chondrosarcoma cell lines treated with YM155 for 48 h. JJ012 and CH2879 show a reduction in G1 and an increase in S phase after treatment with YM155. NDCS1 is not showing a difference in cell-cycle distribution.

No activation of apoptosis was found in chondrosarcoma cell lines after treatment with IC_75_ concentrations of YM155. Caspase 3/7 activation was not observed after 24 or 48 h of treatment (Figure 6a and data not shown). Furthermore, treatment with pan-caspase inhibitor z-VAD could not restore the reduction in viability observed after treatment with YM155 (Figure 6b). PARP expression but not cleaved PARP was observed in CH2879 and NDCS1 cell lines (Figure 6c), however not in SW1353 and only low expression was observed in JJ012.

### YM155 deregulates the cell cycle in a subset of chondrosarcoma cell lines

Because no activation of apoptosis was found in chondrosarcoma cell lines treated with YM155, we evaluated its effect on the cell cycle (Figure 6d). A large increase in S phase and a reduction in G1 were observed in JJ012 cells after 48 h of treatment. CH2879 showed only minor effects in cell-cycle distribution after YM155 treatment and NDCS1 cells did not show any change in cell-cycle distribution after treatment with YM155. Knock down of BIRC5 in JJ012 cells also resulted in a deregulation of the cell cycle (Supplementary Figure 6).

## Discussion

For patients with inoperable or metastatic chondrosarcoma, no treatment options are available resulting in a 10-year survival rate below 30%.^1^ By using a focused RNAi screen targeting core apoptosis machinery components, we identified *BIRC5* as an important player in chondrosarcoma survival and we here show that survivin inhibition using YM155 could be a promising novel treatment strategy for this malignant tumor.

*BIRC5* encodes the survivin protein which is part of the inhibitor of apoptosis family, and is involved in a large variety of cellular processes in the nucleus as well as in the cytoplasm. Nuclear survivin functions in the cell cycle as an essential mitotic regulator being a member of the chromosomal passenger complex. On the other hand, cytoplasmic survivin is predominantly involved in preventing apoptosis.^16^ Overexpression is found in many tumor types including osteosarcomas^22^ and soft tissue sarcomas.^23^ Moreover, survivin upregulation was previously shown in small series of conventional chondrosarcomas.^24, 25, 26^ We here demonstrate high survivin expression in conventional as well as rare chondrosarcoma subtypes in a large panel of >200 chondrosarcomas. Expression was found both in the nucleus and in the cytoplasm in high-grade conventional chondrosarcomas, suggesting a function both in the cell cycle and as an anti-apoptotic protein. In contrast, in dedifferentiated and clear cell chondrosarcomas, survivin was predominantly expressed in the nucleus, suggesting a more prominent role for survivin in cell-cycle regulation.

We also show a positive correlation between nuclear survivin and p53 overexpression in conventional chondrosarcoma. High p53 expression is known to correlate with increasing histological grade in chondrosarcoma^27^ and is suggestive for mutated *TP53*.^28^ Wild-type p53 was shown to be able to repress survivin expression,^17^ which may explain the higher survivin expression in chondrosarcomas with high, probably mutated, p53 expression.

Survivin isoforms are differentially expressed in different types of cancer correlating with survival, depending on the isoform. In chondrosarcoma, we found increased expression of all three isoforms with increasing histological grade. SurvivinΔEx3 has been most extensively studied and has been associated with a worse prognosis in breast, colon and cervical cancer.^29^ In soft tissue sarcomas, all survivin transcripts were strongly overexpressed compared with non-malignant control tissue and elevated expression of survivinΔEx3 was correlated with a worse survival.^30^ Survivin was expressed in normal growth plate tissue, suggesting a role for survivin in endochondral ossification. This is in concordance with the role for survivin described in tissue development and its downregulation with differentiation.^31^ No survivin expression was detected in normal articular cartilage, which is in concordance with the low expression levels observed in other normal adult tissues,^31^ rendering survivin a very attractive therapeutic target.^32^

We here show for the first time that survivin inhibition using YM155 could be a promising novel treatment strategy for chondrosarcoma as YM155 was highly potent in reducing cell viability. YM155 is already in phase I and II clinical trials, so could be readily applicable in clinical trials for chondrosarcoma patients. It is an indirect inhibitor of survivin that blocks the survivin promoter region by binding and disrupting the RNA binding protein ILF3/NF110, thereby preventing survivin transcription.^33^ Another proposed mechanism is disruption of binding of zinc transcription factor SP1 to the survivin promoter by YM155.^34^ Interestingly, *TP53* mutant cell lines were more sensitive to YM155 as compared with *TP53* wt cell lines, suggesting that the *TP53* mutation status could be a predictive biomarker. This was also found in other cancer cell lines.^35^ In *TP53* wt cell lines, mechanisms other than survivin may dominate tumor cell proliferation and survival.

YM155 does not induce apoptosis in the chondrosarcoma cell lines, as shown by the absence of caspase 3 and 7 activity and of PARP cleavage. In previous studies, YM155 was shown to induce apoptosis in leukemia, prostate cancer and breast cancer.^36^ However, in merkel cell carcinoma, YM155 did not induce apoptosis but inhibited DNA synthesis.^37^ Survivin is predominantly expressed during G2/M phase of the cell cycle,^38^ and its inhibition is expected to cause a G2/M arrest. G2/M phase cell-cycle arrest has been described by Lechler *et al.*^25^ in two chondrosarcoma cell lines after siRNA knock down of survivin; however, Yang *et al.*^26^ showed a decrease in S phase after knock down of survivin in the same cells.^26^ We found an increase in S phase after treatment with YM155 in two of our chondrosarcoma cell lines and an increase in G2/M phase in JJ012 cells after BIRC5 knock down. This inconsistency can be caused by several issues including different confluency of the cells, different methods to inhibit survivin and different incubation times before the measurement.

Consistent with our findings, Brun *et al.*^39^ also observed a block in S phase after treatment with YM155 in sonic hedgehog-driven medulloblastoma. We did not observe a difference in cell-cycle distribution in all cell lines, which shows that chondrosarcoma cell lines show a heterogeneous response toward YM155 treatment.

In our hands, combination treatment of YM155 with doxorubicin or cisplatin did not result in synergistic inhibition of viability in two chondrosarcoma cell lines, suggesting that survivin is not involved in chondrosarcoma chemo resistance. However, Lechler *et al.*^25^ previously reported on a synergistic induction of apoptosis after combining survivin siRNA with doxorubicin treatment in two chondrosarcoma cell lines. This can possibly be attributed to the different cell lines and the different mechanism of inhibition used to target survivin.

Phase I and II clinical trials have already been conducted for YM155 as a single agent as well as in combination with chemotherapy for a few tumor types.^36^ Phase I studies show that YM155 is well tolerated; however, larger phase II studies in diffuse large B-cell lymphoma, non-small cell lung cancer, melanoma and prostate cancer do not show very promising results regarding anti-tumor activity.^36^ This does not necessarily mean that this will also be the case for chondrosarcoma, especially since we show here that in chondrosarcoma instead of the induction of apoptosis, YM155 causes a block in the cell cycle, and that patients can be preselected based on *TP53* mutation status. New survivin inhibitors, for example, antisense oligonucleotides and survivin-based vaccines, are in development and some already reached clinical trials.^40^

In conclusion, we show that survivin is essential for chondrosarcoma cell survival and is highly expressed in high-grade chondrosarcomas and absent in normal cartilage. Chondrosarcoma cell lines are highly sensitive for treatment with YM155, especially if *TP53* is mutant, indicating that survivin could be a possible new therapeutic target for patients with chondrosarcoma.

## Materials and methods

### Cell culture

Central conventional chondrosarcoma cell lines SW1353 (ATCC), JJ012,^41^ CH2879,^18^ OUMS27,^42^ CH3573^43^ and L835, and dedifferentiated chondrosarcoma cell lines L3252B, L2975^20^ and NDCS1^44^ were cultured in RPMI-1640 (Gibco, Invitrogen Life Technologies, Scotland, UK) supplemented with 1% penicillin/streptomycin (100 U/ml) and 10 or 20% Fetal Calf Serum (Gibco, Invitrogen Life Technologies). Mesenchymal chondrosarcoma cell line MCS170 was cultured in IMDM medium (Gibco, Invitrogen Life Technologies) supplemented with 1% penicillin/streptomycin (100 U/ml) and 15% Fetal Calf Serum (Supplementary Table 1). All cell lines were cultured at a temperature of 37 °C in a humidified incubator (5% CO_2_). Identity of cell lines was confirmed using the Cell ID GenePrint 10 system (Promega Benelux BV, Leiden, The Netherlands) before and after completion of the experiments. Mycoplasma tests were performed on a regular basis.

### Mutation analysis

Mutations in *TP53*, *IDH1* and *IDH2* were confirmed with Ion AmpliSeqCancer Hotspot Panel v2 (Life Technologies, Thermo Fisher Scientific, Waltham, MA, USA, catalog number 4475346) according to the manufacturer's instructions. Sanger sequencing was performed when validation was necessary (Supplementary Table 1). *TP53* mutations identified were analyzed using prediction software Align GVGD and Sorting Intolerant From Tolerant (SIFT). Also entries in the Catalogue of Somatic Mutations in Cancer (COSMIC) database were analyzed.

### Compounds

The survivin inhibitor YM155 (Catalog No. S1130, Selleckchem, Munich, Germany) and BH3 mimetic ABT-737 (Catalog No. S1002, Selleckchem) were dissolved in DMSO according to the manufacturer's instructions. Doxorubicin and cisplatin were obtained from the in-house hospital pharmacy in a 0.9% NaCl solution. Z-vad-FMK (550377, BD Biosciences San Jose , CA, USA) was used as a general caspase inhibitor and dissolved in DMSO.

### siRNA screen

To identify critical genes for chondrosarcoma cell survival, a focused targeted siRNA screen was performed on the JJ012 central chondrosarcoma cell line targeting 51 apoptosis-related genes (Dharmacon, GE Life Sciences, Landsmeer, the Netherlands) (Supplementary Table 2). In the primary screen, reverse transfection of SMARTpools of four different siRNAs in a final concentration of 50 nm for each gene was performed using DharmaFECT 3 transfection reagent (Thermo Fisher Scientific Inc., T-2003). Deconvolution confirmation screens were performed where each of four individual siRNAs was transfected separately. A gene was considered as a hit when three out of four individual siRNAs mimicked the SMARTpool. *GFP* or *GAPDH* siRNAs were used as negative and *KIF11* siRNAs were used as positive controls. Mock (no siRNA) transfection served as an additional control. The transfection was performed in duplo in u-clear 96-well black clear bottom plates (Corning BV Life Sciences, Amsterdam, The Netherlands) using 7000 cells/well. After 1 day, the medium was replaced. After 5 days, cells were fixed with formalin and stained with Hoechst. Nuclei present in each well were imaged using a BD-pathway microscope (BD Biosciences). Quantification was performed by determining the total Hoechst area using the Image Pro analyzer software (Media Cybernetics, Rockville, MD, USA) and normalizing toward negative controls. Z'factor analysis was performed as a quality control using the mean of si*GFP* and si*GAPDH* as a negative and si*KIF11* as a positive control.

### Immunohistochemistry

Survivin and p53 expression was evaluated in human primary chondrosarcoma tumor tissue using previously constructed tissue microarrays containing 137 conventional chondrosarcomas (92 central of which 42 grade I, 36 grade II, 14 grade III and 45 peripheral including 31 grade I, 11 grade II, 3 grade III)^10^ and 10 chondrosarcoma cell lines. Additionally, survivin expression was evaluated in 20 clear cell, 21 mesenchymal and 25 dedifferentiated chondrosarcomas.^12^ For dedifferentiated chondrosarcoma, the well-differentiated and the dedifferentiated component were scored separately. To visualize survivin, the 71G4B7 antibody (Rabbit mAb #2808, Cell Signaling Technology, Leiden, The Netherlands) was diluted 1:100, and placenta was used as a positive control. p53 expression was determined using an antibody from DAKO (M700101, Heverlee, Belgium) in a 1:800 dilution with tonsil as positive control tissue. Immunohistochemistry was performed according to standard laboratory methods, using citrate (pH 6) as antigen retrieval method, as previously described.^45^ Survivin is known to be expressed in the nucleus as well as in cytoplasm displaying different functions and prognostic significance.^46^ Therefore, nuclear and cytoplasmic expression was scored separately for each core by two independent observers (JVMGB and GA) using a scoring system assessing staining intensity (0=no, 1=weak, 2=moderate, 3=strong) and percentage of staining (0=no, 1=1–24%, 2=25–49%, 3=50–74%, 4=75–100%).^12^ p53 expression was considered as high when added scores were ⩾4, as previously described.^27^ Cores that were lost from the TMA section were excluded from the analysis (*n*=39 for conventional chondrosarcomas, *n*=3 for clear cell chondrosarcoma, *n*=7 for mesenchymal chondrosarcoma and *n*=4 for dedifferentiated chondrosarcoma). Slides were scanned using a Philips intellisite pathology scanner (Philips Healthcare, Brussel, Belgium), and pictures were taken using a Philips IMS viewer (Philips Healthcare).

### RNA isolation and quantitative real-time PCR

RNA was isolated from fresh frozen tissue of 34 conventional chondrosarcoma primary tumor tissues (Supplementary Table 3) and 6 cartilage control tissues, that is, three growth plate, and three articular cartilage tissues. RNA was also isolated from untreated and YM155 treated cell lines. For isolation, TRIzol (Invitrogen, Carlsbad, CA, USA) was used followed by RNA clean up using the RNeasy mini kit (Qiagen, Venlo, The Netherlands) according to the manufacturer's instructions. All patient samples were handled according to the ethical guidelines described in ‘Code for Proper Secondary Use of Human Tissue in The Netherlands' of the Dutch Federation of Medical Scientific Societies. Q-PCR was performed for wild type (wt) survivin, survivin 2b and survivin Δex3 with primers described previously.^47^ Expression was normalized toward housekeeping genes PPIA, CPSF6 and GPR108, as previously described.^48^

### Viability assay

Chondrosarcoma cell lines were plated in 96-well plates so as to achieve 50–70% confluency the following day. Dose response curves were performed using concentrations from 0.01 till 1000 nm. Concentrations used for combination studies were 10 and 50 nm doxorubicin, 250 and 1000 nm cisplatin and 1 and 5 nm YM155, and combinations of drugs were added at the same moment. After 72 h of incubation, a presto blue assay (Life Technologies, Scotland, UK) was performed according to the manufacturer's instructions. Fluorescence was measured reading the plate at 590 nm on a fluorometer (Victor3V, 1420 multilabel counter, Perkin-Elmer, Groningen, the Netherlands). Experiments were performed at least three times in triplicate.

### Apoptosis

To measure apoptosis, the caspase-glo 3/7 assay (Promega, Madison, WI, USA) was used according to the manufacturer's instructions. In brief, cells were plated into white walled 96-well plates (Corning BV Life Sciences, Amsterdam, The Netherlands) and incubated with IC_75_ concentrations of YM155 (determined using 72 h viability assays). After 24 h, the substrate was added in a 1:1 dilution and incubated for 30 min at room temperature. As a positive control, MCS170 cells treated with ABT-737 and doxorubicin were used. Luminescence was measured using a luminometer (Victor3V, 1420 multilabel counter, Perkin-Elmer). Experiments were performed at least two times in duplicate.

### Western blotting

Western blotting for PARP (Cell Signaling Technology) was performed on lysates obtained using hot-SDS buffer (1% SDS, 10 mm Tris/EDTA with complete inhibitor and phosSTOP) as previously described.^9^ For PARP and cleaved PARP detection, Jurkat cell lysates treated with 25 μm etoposide obtained from Cell Signaling Technology (#2043) were used as a positive control. Of each sample, 20 μg protein was loaded on the gel. As a loading control, α-tubulin (clone DM1A, Sigma-Aldrich Chemie BV, Zwijndrecht, The Netherlands) expression was determined. Proteins were blotted onto a PVDF membrane and detected using enhanced chemo luminescence (Pierce ECL Western Blotting Substrate Fisher Scientific, Landsmeer, the Netherlands) followed by exposure of 1 min and development of the film (ECL hyperfilm, Amersham, GE Healthcare, Eindhoven, the Netherlands).

### Cell-cycle analysis

Cells were plated in T25 flasks in amounts ensuring 70% confluency when harvested. For knock down experiments, cells were seeded in 6-well plates. After 48 h of treatment with YM155 IC_50_ concentrations or BIRC5 or GAPDH siRNA, cells were prepared for cell-cycle analysis using methanol and propidium iodide staining as previously described.^49^ Cell-cycle data analysis was performed using WinList 7.1 (Verity Software House, Topsham, ME, USA). Experiments were performed at least two times in duplicate.

### Statistical analysis

Mann–Whitney testing was applied using the Prism 6 GraphPad software (La Jolla, CA, USA) to assess significant differences between survivin expression levels. Bonferroni correction was used to correct for multiple testing. Dose response curves and IC_50_ values were determined using the Prism 6 GraphPad software.
