# Supplementary material for: Targeting survivin as a potential new treatment for chondrosarcoma of bone
Source: Oncogenesis. 2016 May 9;5(5):e222–. doi: 10.1038/oncsis.2016.33 (PMC4945750; doi:10.1038/oncsis.2016.33)
Supplement: Supplementary Figure 1 [file oncsis201633x1.pdf]

Supplementary figure 1

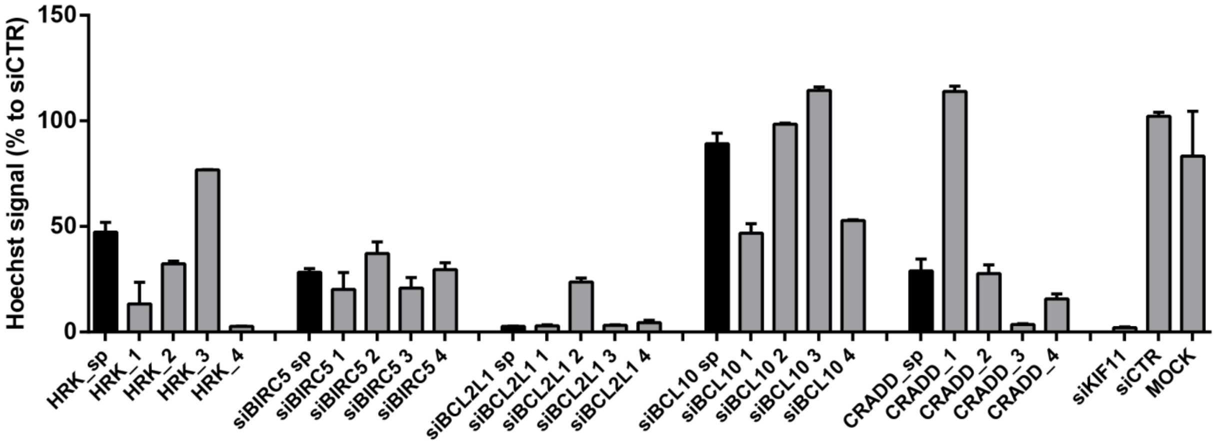

Sfigure 1: Results of deconvolution. Area of cells as a percentage to control siRNA. *BCL2L1*, *BIRC5*, *CRADD* and *HRK* all show 3/4 or 4/4 siRNAs that mimic the smart pool. Data represent means of duplicate values with range. Black bars represent the smart pool and grey bars represent individual siRNAs.
