# Supplementary material for: Targeting survivin as a potential new treatment for chondrosarcoma of bone
Source: Oncogenesis. 2016 May 9;5(5):e222–. doi: 10.1038/oncsis.2016.33 (PMC4945750; doi:10.1038/oncsis.2016.33)
Supplement: Supplementary Figure 2 [file oncsis201633x2.pdf]

**Supplementary figure 2**

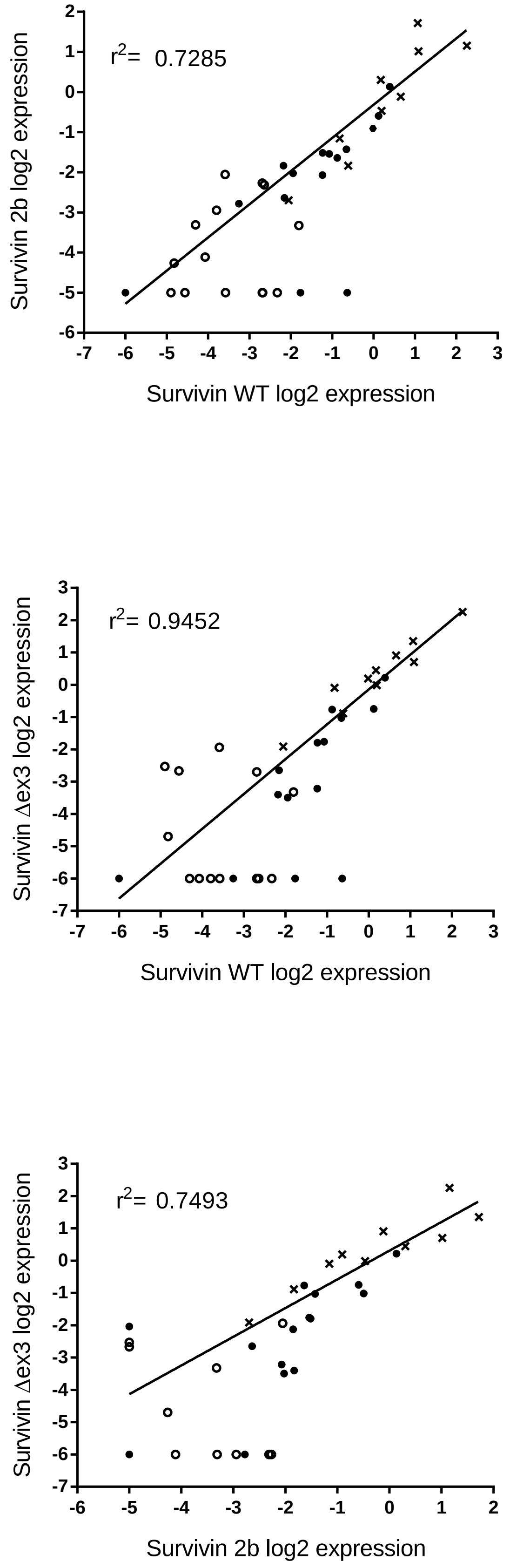

**SFigure 2: Correlation survivin expression A, B, C)** Survivin expression of all three isoforms was correlated in chondrosarcoma tissues and cell lines. Expression is plotted as log2 and $r^2$ was determined to assess correlation. Black dots indicate high grade chondrosarcoma, white dots indicate low grade chondrosarcoma and crosses indicate chondrosarcoma cell lines.
