# Supplementary material for: Targeting survivin as a potential new treatment for chondrosarcoma of bone
Source: Oncogenesis. 2016 May 9;5(5):e222–. doi: 10.1038/oncsis.2016.33 (PMC4945750; doi:10.1038/oncsis.2016.33)
Supplement: Supplementary Figure 3 [file oncsis201633x3.pdf]

Supplementary figure 3

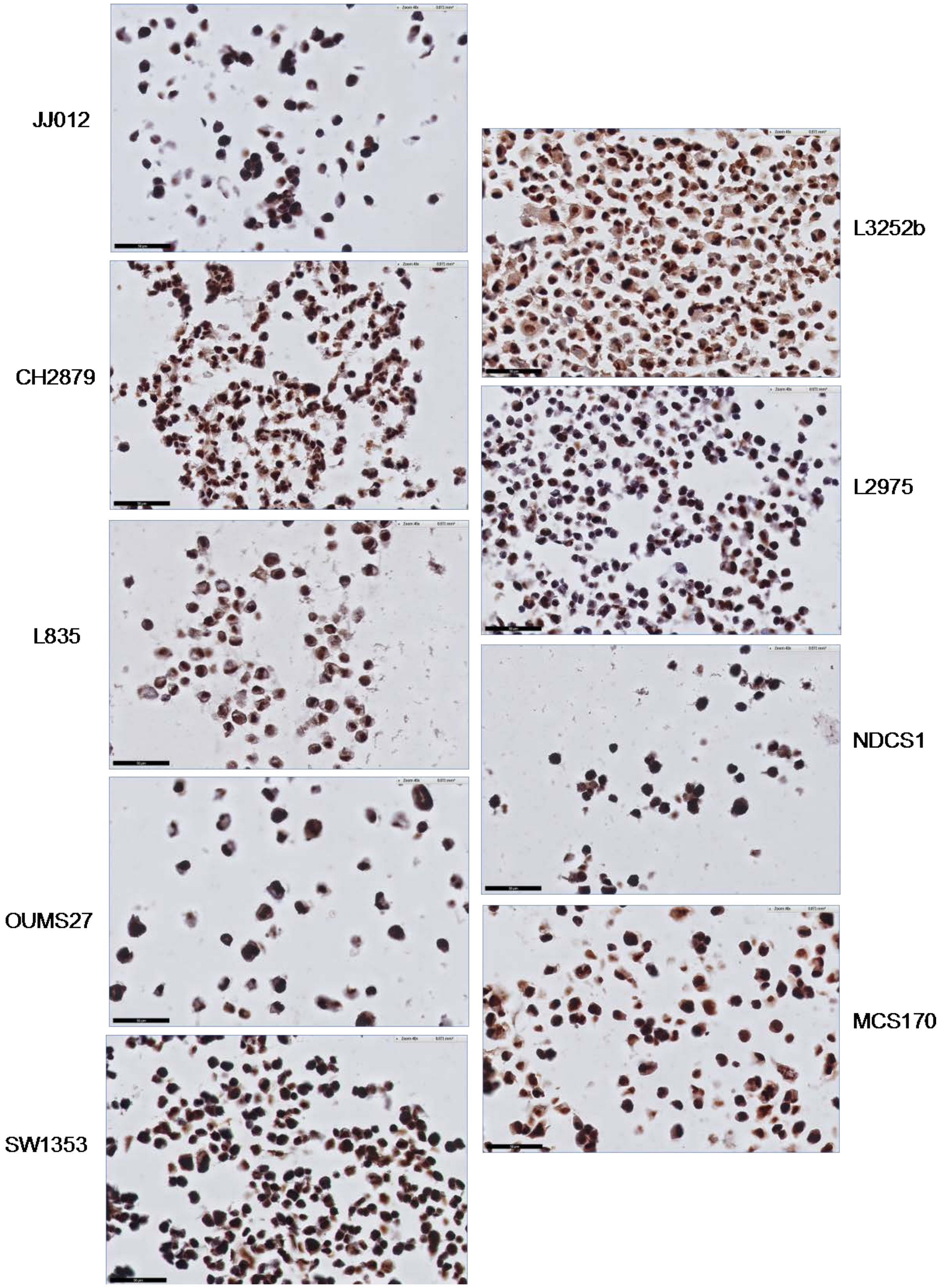

Sfigure 3. Survivin staining in chondrosarcoma cell lines. Survivin is highly expressed in the nucleus of all chondrosarcoma cell lines.
