# Supplementary material for: Targeting survivin as a potential new treatment for chondrosarcoma of bone
Source: Oncogenesis. 2016 May 9;5(5):e222–. doi: 10.1038/oncsis.2016.33 (PMC4945750; doi:10.1038/oncsis.2016.33)
Supplement: Supplementary Figure 4 [file oncsis201633x4.pdf]

Supplementary figure 4

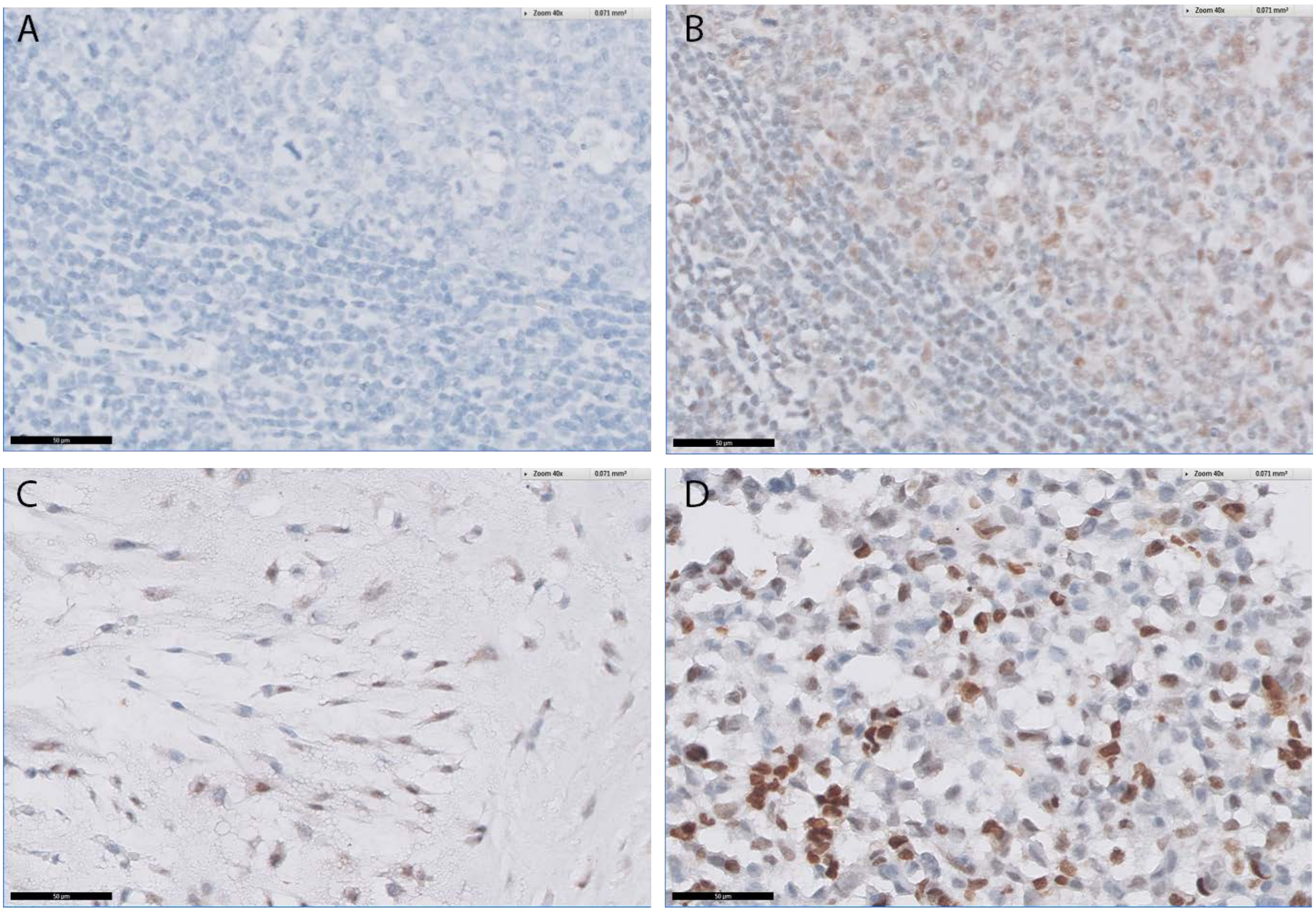

Sfigure 4. Mosaic staining pattern of P53 protein in CH2879. A, B) Negative (A) and positive control (B) tonsil tissue. **C, D)** Mosaic P53 staining in primary tumor tissue (C) and cell pellet (D) of CH2879 cell line.
