# Supplementary material for: Targeting survivin as a potential new treatment for chondrosarcoma of bone
Source: Oncogenesis. 2016 May 9;5(5):e222–. doi: 10.1038/oncsis.2016.33 (PMC4945750; doi:10.1038/oncsis.2016.33)
Supplement: Supplementary Figure 5 [file oncsis201633x5.pdf]

**Supplementary figure 5**

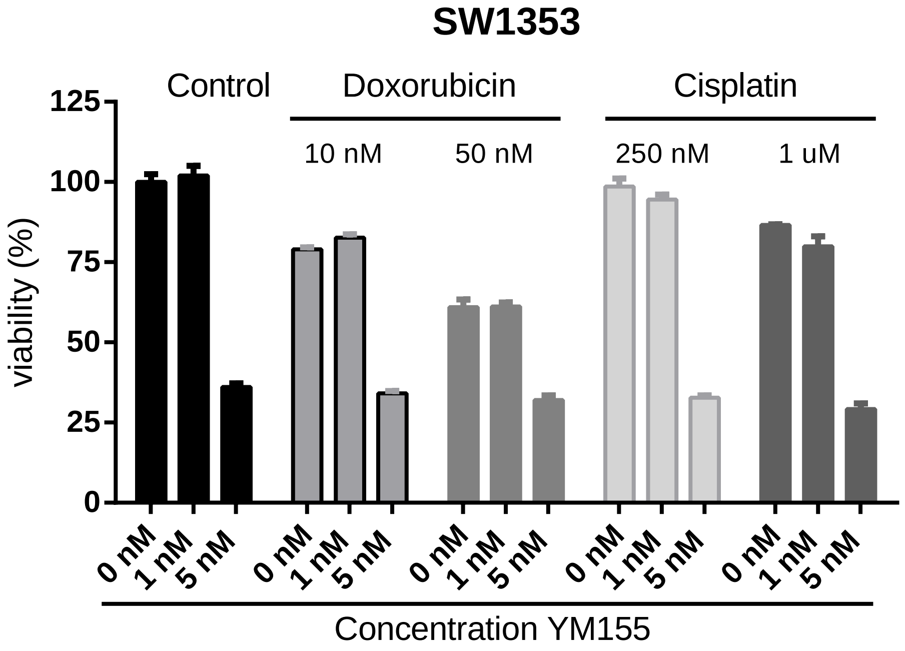


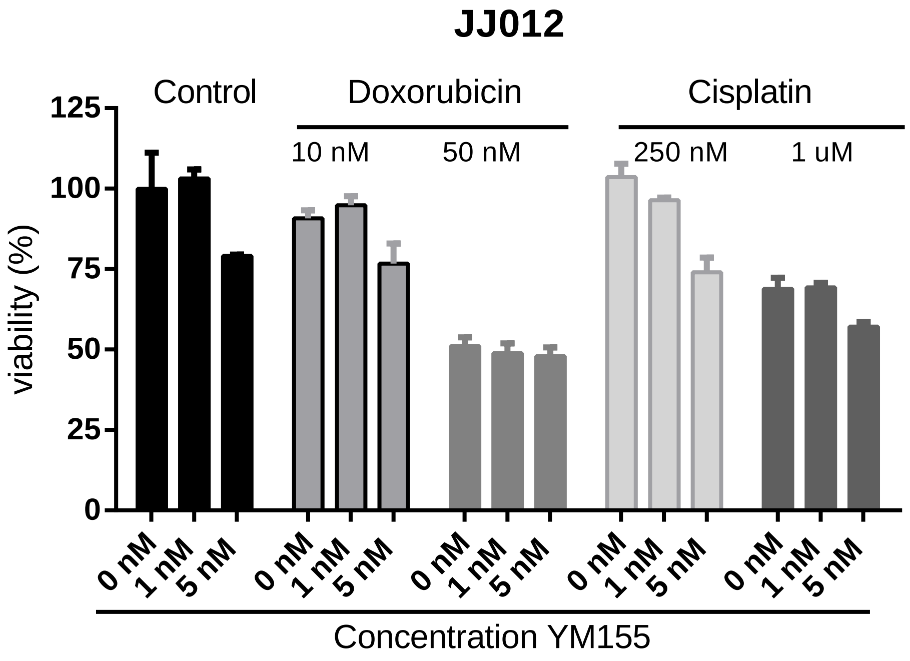


**SFigure 5. No synergistic effects of YM155 combined with doxorubicin or cisplatin are observed in JJ012 or SW1353 cell lines** as determined by presto blue assay. Values represent percentages to non-treated control cells.
