# Supplementary material for: Targeting survivin as a potential new treatment for chondrosarcoma of bone
Source: Oncogenesis. 2016 May 9;5(5):e222–. doi: 10.1038/oncsis.2016.33 (PMC4945750; doi:10.1038/oncsis.2016.33)
Supplement: Supplementary Figure 6 [file oncsis201633x6.pdf]

**Supplementary figure 6**

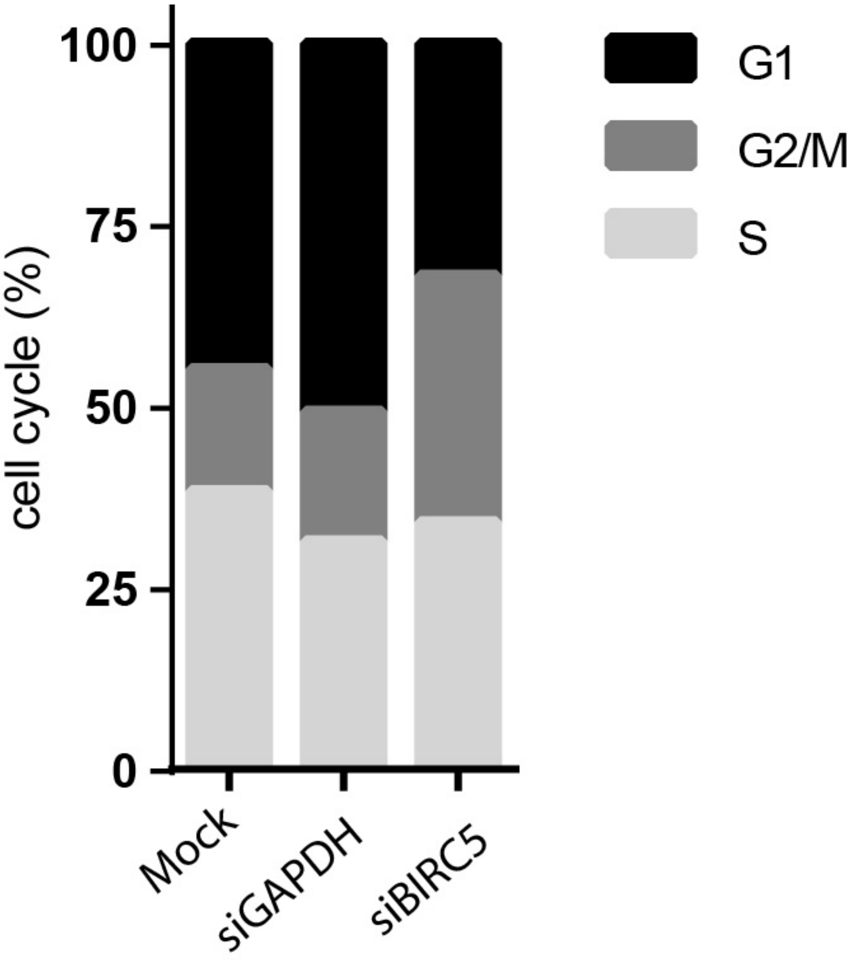


**SFigure 6. BIRC5 knock down in JJ012 cells results in an increase in G2/M phase.** Cell cycle analysis performed after knock down of GAPDH or BIRC5 in chondrosarcoma cells. Results shown are representative of a duplicate measurement.
