# Supplementary material for: Targeting survivin as a potential new treatment for chondrosarcoma of bone
Source: Oncogenesis. 2016 May 9;5(5):e222–. doi: 10.1038/oncsis.2016.33 (PMC4945750; doi:10.1038/oncsis.2016.33)
Supplement: Supplementary Table 1 [file oncsis201633x7.pdf]

Supplementary Table 1

Cell line	Subtype	*TP53* status	Prediction *Align GVGD*	*SIFT*	Hits in Cosmic	P53 staining *Intensity*	percentage	*IDH1* or *-2* status	Ref
**JJ012**	CCS	p.Gly199Val	Pathogenic	Deleterious	10	Strong	100%	*IDH1* p.Arg132Gly	Scully et al. 2000
**SW1353**	CCS	p.Val203Leu	Tolerated	Tolerated	5	Strong	100%	*IDH2* p.Arg172Ser	ATCC
**CH3573**	CCS	p.Leu201Cysfs*46	Pathogenic	Deleterious	3	No	0%	WT	Calabuig-Farinas et al. 2012
**OUMS27**	CCS	p.Ser149Tyrfs*31	Pathogenic	deleterious	0	No	0%	WT	Kunisada et al. 1998
**L835**	CCS	WT				moderate	70%	*IDH1* p.Arg132Cys	Van Oosterwijk et al. 2012
**CH2879**	CCS	p.Ser366Ala<br>p.Arg273Cys#	Uncertain<br>Pathogenic	Tolerated<br>Deleterious	2<br>628	strong	30-40%	WT	Gil Benso et al. 2003
**NDCS1**	Dedif	p.Cys242Ser	Pathogenic	Deleterious	3	strong	100%	WT	Kudo et al. 2007
**L2975**	Dedif	p.Cys229Serfs*7	Pathogenic	Deleterious	0	Weak	5%	*IDH2* p.Arg172Trp	Van Oosterwijk et al. 2012
**L3252B**	Dedif	WT				Moderate	5%	WT	Van Oosterwijk et al. 2012
**MCS-170**	Mes	WT				no	0%	WT	

#only found in 17% of cells

Stable 1: Chondrosarcoma cell line panel. Chondrosarcoma cell line panel used to study the effect of survivin inhibition using YM155. *TP53* and *IDH* mutation status are indicated. Align GVDG and SIFT prediction tools were used to predict the functional consequence of the identified *TP53* mutation. Mutations were searched in COSMIC to determine previous identifications. P53 staining intensity and percentage are indicated. CCS= Conventional chondrosarcoma, Dedif= Dedifferentiated chondrosarcoma, Mes=Mesenchymal chondrosarcoma.
