# Supplementary material for: Targeting survivin as a potential new treatment for chondrosarcoma of bone
Source: Oncogenesis. 2016 May 9;5(5):e222–. doi: 10.1038/oncsis.2016.33 (PMC4945750; doi:10.1038/oncsis.2016.33)
Supplement: Supplementary Table 2 [file oncsis201633x8.pdf]

Supplementary table 2

Well	Gene Symbol	Gene Id	Accession Number	GI Number	Sequence
A02	*CASP1*	834	NM_033295	73622117	CCGCAAGGUUCGAUUUUCA
A02	*CASP1*	834	NM_033295	73622117	GAAUAUGCCUGUUCCUGUG
A02	*CASP1*	834	NM_033295	73622117	GACUCAUUGAACAUAUGCA
A02	*CASP1*	834	NM_033295	73622117	AGACAUCCCACAAUGGGCU
A03	*CASP10*	843	NM_032974	98985797	GAAAUGACCUCCCUAAGUU
A03	*CASP10*	843	NM_032974	98985797	GAAGGCAGCUGGUAUAUUC
A03	*CASP10*	843	NM_032974	98985797	CAAAGGGUUUCUCUGUUUA
A03	*CASP10*	843	NM_032974	98985797	ACAAUAAUGUGACGAAAGU
A04	*CASP14*	23581	NM_012114	6912285	GGACAUAUCUUGGAACUUC
A04	*CASP14*	23581	NM_012114	6912285	ACAGAUGCCUUGCACGUUU
A04	*CASP14*	23581	NM_012114	6912285	GAGCUAAGCCCAAGGUGUA
A04	*CASP14*	23581	NM_012114	6912285	GAGAUGAGAUUGUGAUGGU
A05	*CASP2*	835	NM_032983	39995060	GAACGCACUUAUCAAGGAU
A05	*CASP2*	835	NM_032983	39995060	GGAGAGUGAUGCCGGUAAA
A05	*CASP2*	835	NM_032983	39995060	GCACUGGUGUUGAGCAAUG
A05	*CASP2*	835	NM_032983	39995060	UGACGUCCAUGUUCUAUGU
A06	*CASP3*	836	NM_032991	73622122	CCGACAAGCUUGAAUUUAU
A06	*CASP3*	836	NM_032991	73622122	GGUUAUUAUUCUUGGCGAA
A06	*CASP3*	836	NM_032991	73622122	UGAGAUGGGUUUAUGUAUA
A06	*CASP3*	836	NM_032991	73622122	GAUGCGUGAUGUUUCUAAA
A07	*CASP4*	837	NM_001225	73622123	GGACUAUAGUGUAGAUGUA
A07	*CASP4*	837	NM_001225	73622123	GAGGGAAUCUGCGGAACUG
A07	*CASP4*	837	NM_001225	73622123	GAGACUAUGUAAAGAAAGA
A07	*CASP4*	837	NM_001225	73622123	UAGAGGAAGUAUUUCGGAA
A08	*CASP6*	839	NM_032992	73622127	CAUGGUACAUUCAAGAUUU
A08	*CASP6*	839	NM_032992	73622127	AAAUAUGGCUCCUCCUUAG
A08	*CASP6*	839	NM_032992	73622127	CAACAUAACUGAGGUGGAU
A08	*CASP6*	839	NM_032992	73622127	GUUCAAAGGAGACAAGUGU
A09	*CASP7*	840	NM_033340	73623014	GGGCAAAUGCAUCAUAAUA
A09	*CASP7*	840	NM_033340	73623014	GAACUCUACUUCAGUCAAU
A09	*CASP7*	840	NM_033340	73623014	UACCGUCCCUCUUCAGUAA
A09	*CASP7*	840	NM_033340	73623014	CCAGACCGGUCCUCGUUUG
A10	*CASP8*	841	NM_033358	122056472	GGACAAAGUUUACCAAAUG
A10	*CASP8*	841	NM_033358	122056472	GAAGAUAAUCAACGACUAU
A10	*CASP8*	841	NM_033358	122056472	CAACGACUAUGAAGAAUUC
A10	*CASP8*	841	NM_033358	122056472	GCAAGAACCCAUCAAGGAU
A11	*CASP9*	842	NM_032996	14790127	GCUUCCAGAUUGACGACAA
A11	*CASP9*	842	NM_032996	14790127	CCAGGCAGCUGAUCAUAGA
A11	*CASP9*	842	NM_032996	14790127	GGACAUGCUGGCUUCGUUU
A11	*CASP9*	842	NM_032996	14790127	GGACUUUGCAGCCGGCACA
B02	*BCL10*	8915	NM_003921	119466529	GAAGAUUACAGAUGAAGUG
B02	*BCL10*	8915	NM_003921	119466529	GCCACCAGAUCUACAGUUA
B02	*BCL10*	8915	NM_003921	119466529	GAAAUUUCUUGUCGAACAU
B02	*BCL10*	8915	NM_003921	119466529	GGGCAUCCACUGUCAUGUA
B03	*BCL2*	596	NM_000657	72198345	GGAGGAUUGUGGCCUUCUU
B03	*BCL2*	596	NM_000657	72198345	GGGAGAUAGUGAUGAAGUA
B03	*BCL2*	596	NM_000657	72198345	UCAUGUGUGUGGAGAGCGU
B03	*BCL2*	596	NM_000657	72198345	GAAGUACAUCCAUUAUAAG
B04	*BCL2A1*	597	NM_004049	14574570	GGAAAGAUCUGUGAAAUGC
B04	*BCL2A1*	597	NM_004049	14574570	GAAGGUAUUCUCAUCAAGA
B04	*BCL2A1*	597	NM_004049	14574570	CCUAUAAGGAGAUUUCAUA

B04	*BCL2A1*	597	NM_004049	14574570	UGACUGAUAUGGAAACGAU
B05	*BCL2L1*	598	NM_001191	20336333	CCUACAAGCUUUCCCAGAA
B05	*BCL2L1*	598	NM_001191	20336333	GGAGGCAGGCGACGAGUUU
B05	*BCL2L1*	598	NM_001191	20336333	GAUUCAGGCUGCUUGGGAU
B05	*BCL2L1*	598	NM_001191	20336333	GAAAGGGCCAGGAACGCUU
B06	*BCL2L10*	10017	NM_020396	20336328	UCUCUGGACACGAUUAUUA
B06	*BCL2L10*	10017	NM_020396	20336328	AGAGUGGUGACGCUCGUGA
B06	*BCL2L10*	10017	NM_020396	20336328	GGAGCAGGAGGGCGACGUC
B06	*BCL2L10*	10017	NM_020396	20336328	GGAACCGCUUCGAGCUGGU
B07	*BCL2L11*	10018	NM_207002	116734658	CCGAGAAGGUAGACAAUUG
B07	*BCL2L11*	10018	NM_207002	116734658	AUGUAAGUUCUGAGUGUGA
B07	*BCL2L11*	10018	NM_207002	116734658	GUUCUGAGUGUGACCGAGA
B07	*BCL2L11*	10018	NM_207002	116734658	UGAUGUAAGUUCUGAGUGU
B08	*BCL2L14*	79370	NM_030766	13540528	GAAAUUCCCAAUCCAGUGA
B08	*BCL2L14*	79370	NM_030766	13540528	GUCAAAGGACGUUGGAAUA
B08	*BCL2L14*	79370	NM_030766	13540528	UAACUGAGGGUCUCUCCUU
B08	*BCL2L14*	79370	NM_030766	13540528	GGAAUUGUUCAGCAAAUGA
B09	*BCL2L2*	599	NM_004050	157266332	GGAAGAGUGAGCAGGACAC
B09	*BCL2L2*	599	NM_004050	157266332	CAUAAGUGCUGAUCUAGAA
B09	*BCL2L2*	599	NM_004050	157266332	GCAUAUGGGUUCUAGAGUA
B09	*BCL2L2*	599	NM_004050	157266332	GUUAUAAGCUGAGGCAGAA
B10	*BAD*	572	NM_004322	14670386	CAGAGUUUGAGCCGAGUGA
B10	*BAD*	572	NM_004322	14670386	GUUCCAGAUCCCAGAGUUU
B10	*BAD*	572	NM_004322	14670386	GCAACGCAGAUGCGGCAAA
B10	*BAD*	572	NM_004322	14670386	GUGACGAGUUUGUGGACUC
B11	*BAG1*	573	NM_004323	124494250	GCGAAGAGAUGAAUCGGAG
B11	*BAG1*	573	NM_004323	124494250	GGGCAGCAGUGAACCAGUU
B11	*BAG1*	573	NM_004323	124494250	ACACUGAUCCUGCCAGAAA
B11	*BAG1*	573	NM_004323	124494250	CAAUAGAGCAGUUUAUGAA
C02	*BAG4*	9530	NM_004874	14574569	GUUAUACUCAGACCAGUUA
C02	*BAG4*	9530	NM_004874	14574569	GAUAUCCGCCUUCACAGAA
C02	*BAG4*	9530	NM_004874	14574569	UAUCCUAGCUACAAUUCUA
C02	*BAG4*	9530	NM_004874	14574569	GAGAAGGUCCAGUAUCUUG
C03	*BAG5*	9529	NM_001015048	62548853	GAAGAAGGCAUCCAAGAUA
C03	*BAG5*	9529	NM_001015048	62548853	GAAAUCUGAUGAAUGGGAG
C03	*BAG5*	9529	NM_001015048	62548853	CAACAUCCUUCUAUUAGUA
C03	*BAG5*	9529	NM_001015048	62548853	GAAAUUAUCGGAGGGAGGU
C04	*BAK1*	578	NM_001188	109698605	CAGAGAAUGCCUAUGAGUA
C04	*BAK1*	578	NM_001188	109698605	GCUUCGUGGUCGACUUCAU
C04	*BAK1*	578	NM_001188	109698605	CAACCGACGCUAUGACUCA
C04	*BAK1*	578	NM_001188	109698605	CGACAUCAACCGACGCUAU
C05	*BAX*	581	NM_138763	163659849	CUGAGCAGAUCAUGAAGAC
C05	*BAX*	581	NM_138763	163659849	GAACUGAUCAGAACCAUCA
C05	*BAX*	581	NM_138763	163659849	ACAUGUUUUCUGACGGCAA
C05	*BAX*	581	NM_138763	163659849	CAUUGGACUUCCUCCGGGA
C06	*BBC3*	27113	NM_014417	24475588	CCGAGAUGGAGCCCAAUUA
C06	*BBC3*	27113	NM_014417	24475588	CGGACGACCUCAACGCACA
C06	*BBC3*	27113	NM_014417	24475588	CCUGGAGGGUCCUGUACAA
C06	*BBC3*	27113	NM_014417	24475588	GGCGGAGACAAGAGGAGCA
C07	*BMF*	90427	NM_001003943	51558703	GAACCAAAAUCGUGUGUGG
C07	*BMF*	90427	NM_001003943	51558703	AAUCGUGUGUGGUGGCAGA
C07	*BMF*	90427	NM_001003943	51558703	GAGUAACAGAUAACGAUUA
C07	*BMF*	90427	NM_001003943	51558703	GAAUCUACCAGUUGUCGAA
C08	*BOK*	666	NM_032515	34335395	GGAGCGCGCCCGAGCGUGC

C08	*BOK*	666	NM_032515	34335395	GGCCCAGCGUCUACCGCAA
C08	*BOK*	666	NM_032515	34335395	AGAUCAUGGACGCCUUUGA
C08	*BOK*	666	NM_032515	34335395	GAUGGACUGAUGUCCUCAA
C09	*MCL1*	4170	NM_182763	33519457	GGACCAACUACAAAUUAAU
C09	*MCL1*	4170	NM_182763	33519457	GCUACGUAGUUCGGGCAAA
C09	*MCL1*	4170	NM_182763	33519457	CGAAGGAAGUAUCGAAUUU
C09	*MCL1*	4170	NM_182763	33519457	AGAACGAAUUGAUGUGUAA
C10	*BID*	637	NM_197967	37574727	GCCAGAAGCUACUGCGAUG
C10	*BID*	637	NM_197967	37574727	UGCAAUACAUACCACGCUA
C10	*BID*	637	NM_197967	37574727	GCACCUACGUGAGGAGCUU
C10	*BID*	637	NM_197967	37574727	GAGUAAGGGCACUGACGGA
C11	*HRK*	8739	NM_003806	4504492	CUGCUCGGCAGGCGGAACU
C11	*HRK*	8739	NM_003806	4504492	UAGGCGACGAGCUGCACCA
C11	*HRK*	8739	NM_003806	4504492	GCUCAAGGCGCUAGGCGAC
C11	*HRK*	8739	NM_003806	4504492	UCAAGGCGCUAGGCGACGA
D02	*PMAIP1*	5366	NM_021127	148922873	AAACUGAACUUCCGGCAGA
D02	*PMAIP1*	5366	NM_021127	148922873	AAUCUGAUAUCCAAACUCU
D02	*PMAIP1*	5366	NM_021127	148922873	CUGGAAGUCGAGUGUGCUA
D02	*PMAIP1*	5366	NM_021127	148922873	GCAAGAACGCUCAACCGAG
D03	*BIK*	638	NM_001197	21536418	CCAUGGAGGUUCUUGGCAU
D03	*BIK*	638	NM_001197	21536418	AAGAGGACCUGGACCCUAU
D03	*BIK*	638	NM_001197	21536418	GCACCUGCUGCUCAAGUGA
D03	*BIK*	638	NM_001197	21536418	GGAGGGCAGUGACGCAUUG
D04	*API5*	8539	NM_006595	5729729	CAAGGUUUGUGACAUAUUU
D04	*API5*	8539	NM_006595	5729729	GAAAGGUGGUACUAAGGAA
D04	*API5*	8539	NM_006595	5729729	GAACAAGAUUAAAGUCGUU
D04	*API5*	8539	NM_006595	5729729	AAAGAUGCCUAUCAAGUGA
D05	*BIRC1*	4671	NM_022892	119393875	CAAGGGAUUUGUUGACAUA
D05	*BIRC1*	4671	NM_022892	119393875	GUACAAGAUUCCCUGUCUA
D05	*BIRC1*	4671	NM_022892	119393875	GUAAAGAGCUAUAUGGAUA
D05	*BIRC1*	4671	NM_022892	119393875	GAAAUUACCCAGUAUAUUC
D06	*BIRC2*	329	NM_001166	41349435	UAUAGGACCUGGAGAUAGG
D06	*BIRC2*	329	NM_001166	41349435	GAAACAAACAGUUCAAAGU
D06	*BIRC2*	329	NM_001166	41349435	CAAUUGACAUGUGUGCUUC
D06	*BIRC2*	329	NM_001166	41349435	GAACAGAAUGGCUCUCUUU
D07	*BIRC3*	330	NM_001165	33946283	CAUGUGAACUGUACCGAAU
D07	*BIRC3*	330	NM_001165	33946283	GAAGUGUCCAUAGUGUUUA
D07	*BIRC3*	330	NM_001165	33946283	UCAAUGAUCUUGUGUUAGA
D07	*BIRC3*	330	NM_001165	33946283	GAUGAAUACUCCUGUGAUU
D08	*BIRC5*	332	NM_001012270	59859879	CAAAGGAAACCAACAAUAA
D08	*BIRC5*	332	NM_001012270	59859879	GCAAAGGAAACCAACAAUA
D08	*BIRC5*	332	NM_001012270	59859879	CACCGCAUCUCUACAUUCA
D08	*BIRC5*	332	NM_001012270	59859879	CCACUGAGAACGAGCCAGA
D09	*BIRC6*	57448	NM_016252	153792693	CCAAAGAUACGGUUACAUA
D09	*BIRC6*	57448	NM_016252	153792693	GGUCAAAGAUCACUUAGUA
D09	*BIRC6*	57448	NM_016252	153792693	GCAACGAUGUGCCAUGUUA
D09	*BIRC6*	57448	NM_016252	153792693	ACAAGCACCUCUCGCAUUA
D10	*DIABLO*	56616	NM_019887	42544195	GCAGAUCAGGCCUCUAUAA
D10	*DIABLO*	56616	NM_019887	42544195	CCGACAAUAUACAAGUUUA
D10	*DIABLO*	56616	NM_019887	42544195	GGAAACCACUUGGAUGACU
D10	*DIABLO*	56616	NM_019887	42544195	UAGAAGAGCUCCGUCAGAA
D11	*BIRC4*	331	NM_001167	32528298	GAGGAACCCUGCCAUGUAU
D11	*BIRC4*	331	NM_001167	32528298	GCACGGAUCUUUACUUUUG
D11	*BIRC4*	331	NM_001167	32528298	GAACUGGGCAGGUUGUAGA

D11	*BIRC4*	331	NM_001167	32528298	GAGGAGGGCUAACUGAUUG
E02	*PDCD8*	9131	NM_004208	22202627	GGUAGAAACUGACCACAUA
E02	*PDCD8*	9131	NM_004208	22202627	CAAGGAAGAUCAUUAAGGA
E02	*PDCD8*	9131	NM_004208	22202627	GCAUGAAGAUCUCAAUGAA
E02	*PDCD8*	9131	NM_004208	22202627	GAACAUCUUUAACCGAAUG
E03	*APAF1*	317	NM_181869	32483362	GAACAAGCUCAUAGUAUUA
E03	*APAF1*	317	NM_181869	32483362	GUAUGAUGGAGCUGCAAAU
E03	*APAF1*	317	NM_181869	32483362	AGAUAAUGAUUCCUACGUA
E03	*APAF1*	317	NM_181869	32483362	CAGUAAUGGGUCCUAAAUA
E04	*AVEN*	57099	NM_020371	56699476	UAACUGGGAUCGAUAUCAA
E04	*AVEN*	57099	NM_020371	56699476	GAUUAGGGAUGCAGUUAAA
E04	*AVEN*	57099	NM_020371	56699476	GAACAGGGAAAUUAUUCUA
E04	*AVEN*	57099	NM_020371	56699476	GUUAUUGGUUCGAGCCCUU
E05	*BFAR*	51283	NM_016561	7706090	CAUCAUCAAUGCUAUGUUA
E05	*BFAR*	51283	NM_016561	7706090	ACGAGUAAAUGGAAGGUUG
E05	*BFAR*	51283	NM_016561	7706090	GCUACGACAUCCUGGUUAA
E05	*BFAR*	51283	NM_016561	7706090	CAAGACGCCCUAUACCAUA
E06	*CFLAR*	8837	NM_003879	21361768	UAAAGAACAUCCACAGAAU
E06	*CFLAR*	8837	NM_003879	21361768	AAUAACUUCAGGCUCCAUA
E06	*CFLAR*	8837	NM_003879	21361768	GAUGUGUCCUCAUUAAUUU
E06	*CFLAR*	8837	NM_003879	21361768	GCUAUGAAGUCCAGAAAUU
E07	*CRADD*	8738	NM_003805	51988883	ACAAUGCUCCUGCUGGAUA
E07	*CRADD*	8738	NM_003805	51988883	UAGAUUCCCUACAGGAGUU
E07	*CRADD*	8738	NM_003805	51988883	ACGGAUAUCUACCGCUGUA
E07	*CRADD*	8738	NM_003805	51988883	GGCCAGAGACAAACAAGUA
E08	*CYCS*	54205	NM_018947	34328939	GGAAGAAAGGGCAGACUUA
E08	*CYCS*	54205	NM_018947	34328939	GCAUUAAGAAGAAGGAAGA
E08	*CYCS*	54205	NM_018947	34328939	ACACAGCCGCCAAUAAGAA
E08	*CYCS*	54205	NM_018947	34328939	GCACAAGACUGGGCCAAAU
E09	*DAXX*	1616	NM_001350	53828721	GGAGUUGGAUCUCUCAGAA
E09	*DAXX*	1616	NM_001350	53828721	GGAAACAGCUAUGUGGAAA
E09	*DAXX*	1616	NM_001350	53828721	CUACAGAUCUCCAAUGAAA
E09	*DAXX*	1616	NM_001350	53828721	GCUACAAGCUGGAGAAUGA
E10	*DEDD*	9191	NM_001039712	89353286	GGACGUGACUUCUUAUUGG
E10	*DEDD*	9191	NM_001039712	89353286	GGCAAUGUCUUCUCUAACA
E10	*DEDD*	9191	NM_001039712	89353286	UCACCUACCUCGAUGCAUU
E10	*DEDD*	9191	NM_001039712	89353286	CUACAUCAAUGGCUCUUUA
E11	*TOSO*	9214	NM_005449	34147517	GCAAGAAUCUGUUCCUAGU
E11	*TOSO*	9214	NM_005449	34147517	GCAAUACCCACGCAAGAAU
E11	*TOSO*	9214	NM_005449	34147517	GGUUUCAUCUGCCCUAUUU
E11	*TOSO*	9214	NM_005449	34147517	GAAUACAAGGGCCGAGUUA
F02	*MOAP1*	64112	NM_022151	73747827	GAAGAGAAGGCGAUUGCUA
F02	*MOAP1*	64112	NM_022151	73747827	GAGGAGCAAUUGAGAGAGA
F02	*MOAP1*	64112	NM_022151	73747827	AGGAAAAGUUGUCGGCUUA
F02	*MOAP1*	64112	NM_022151	73747827	GGAAAGCGCUAUUGAUUGC

STable 2: siRNAs in apoptosis screen. All siRNAs present in the apoptosis screen are shown as well as gene id, asseccion number, GI number and sequence.
