# Supplementary material for: Targeting survivin as a potential new treatment for chondrosarcoma of bone
Source: Oncogenesis. 2016 May 9;5(5):e222–. doi: 10.1038/oncsis.2016.33 (PMC4945750; doi:10.1038/oncsis.2016.33)
Supplement: Supplementary Table 3 [file oncsis201633x9.pdf]

Supplementary table 3

Sample	Grade	IDH Mutation	Amount
Tumour	Low	*IDH1*<br>*IDH2*<br>Wild type	14<br>1<br>4
	High	*IDH1*<br>*IDH2*<br>Wild type	6<br>4<br>5
Growth plate	Not applicable	N/A	3
Cartilage	Not applicable	N/A	3

Stable 3: Patient samples subjected to survivin expression analysis by Q-PCR. Information of RNA samples used to evaluate survivin expression. Shown are grade, mutation status and amounts.
